# Primary Immune Deficiencies – Principles of Care

**DOI:** 10.3389/fimmu.2014.00627

**Published:** 2014-12-15

**Authors:** Helen Chapel, Johan Prevot, Hubert Bobby Gaspar, Teresa Español, Francisco A. Bonilla, Leire Solis, Josina Drabwell, The Editorial Board for Working Party on Principles of Care at IPOPI

**Affiliations:** ^1^University of Oxford, Oxford, UK; ^2^International Patient Organisation for Primary Immunodeficiencies (IPOPI), Downderry, UK; ^3^University College London Institute of Child Health, London, UK; ^4^Hospital General Vall d’Hebron, Barcelona, Spain; ^5^Boston Children’s Hospital, Harvard Medical School, Boston, MA, USA

**Keywords:** primary immunodeficiencies, awareness, diagnosis, management, treatments, worldwide

## Abstract

Primary immune deficiencies (PIDs) are a growing group of over 230 different disorders caused by ineffective, absent or an increasing number of gain of function mutations in immune components, mainly cells and proteins. Once recognized, these rare disorders are treatable and in some cases curable. Otherwise untreated PIDs are often chronic, serious, or even fatal. The diagnosis of PIDs can be difficult due to lack of awareness or facilities for diagnosis, and management of PIDs is complex. This document was prepared by a worldwide multi-disciplinary team of specialists; it aims to set out comprehensive principles of care for PIDs. These include the role of specialized centers, the importance of registries, the need for multinational research, the role of patient organizations, management and treatment options, the requirement for sustained access to all treatments including immunoglobulin therapies and hematopoietic stem cell transplantation, important considerations for developing countries and suggestions for implementation. A range of healthcare policies and services have to be put into place by government agencies and healthcare providers, to ensure that PID patients worldwide have access to appropriate and sustainable medical and support services.

## Introduction

### Why a principles of care document/call to action

Primary immune deficiencies (PIDs) are a large and growing group of over 230 different disorders, caused when some components of the immune system (mainly cells and proteins) are defective. While PIDs are generally recognized as rare disorders, some are more common than others. Taken as a whole, they represent an important group of conditions that, if not treated, can be chronic, life-long, serious, and even fatal. The lives of patients with PIDs are profoundly impacted by their condition. The immune system normally helps the body to fight infections caused by germs (or “micro-organisms”) such as bacteria, viruses, fungi, and protozoa. Owing to defective immune systems, people with PIDs are more prone to infections. In addition, a poorly regulated immune system may start to attack tissues, leading to inflammation, and autoimmunity (1, 2). When PIDs are left undiagnosed or are misdiagnosed, chronic illness and disability take a heavy toll on healthcare resources (3, 4).

The immune system is divided into two parts, each of which contains two components: on the one hand, soluble proteins may be particular for one germ (antibodies) or non-specific (complement). The other components are cellular – those that are specific for one germ only (lymphocytes) and innate cells that are involved in clearing all types of infections (such as phagocytes including macrophages and neutrophils).

Primary immune deficiencies are currently classified into groups, depending on the part(s) of the immune system affected. Over half the affected patients have antibody deficiencies and their treatment consists of replacing the missing antibodies (5). Cellular defects of lymphocytes are more severe and require replacement of stem cells that can mature to effective immune blood cells [hematopoietic stem cell transplantation (HSCT)] (6, 7) or replacement of the faulty gene.

While it is considered that many PIDs can be diagnosed easily with two simple blood tests (8), unfortunately many PIDs remain undiagnosed due to failure to consider this diagnosis. In addition, access to treatment varies widely between different regions of the world (9) and even from country to country within the same continent.

In order to tackle these disparities and ensure early diagnosis and appropriate access to treatments for all PID patients worldwide, a range of healthcare policies and services need to be put into place. While some countries have managed to do this, most have not.

A worldwide multi-disciplinary team of specialists prepared this document, which sets out comprehensive principles of care for PIDs. These include the role of specialized centers, the importance of registries, the need for multinational research, the role of patient organizations, the need for sustained access to all treatments, including immunoglobulin (Ig) therapies and HSCT, worldwide.

It is hoped that these principles of care will guide stakeholders and decision-makers in a common goal to ensure that all PID patients can access the care they are entitled to in order to live normal and productive lives. There are examples of national and international networks in other rare diseases that provide opportunities for collaborations to improve patient care, an example being hemophilia. Such networks are fruitful and although already underway in some areas, provision of sustainable infra-structures is important to overcome major obstacles in the care of PID patients throughout the world.

### Brief PIDs overview: Rare and chronic

Primary immune deficiencies are rare diseases of relatively recent description. Infections were a common cause of death in the general population up to the first half of the nineteenth century, so these diseases were not suspected. With the advent of improved hygiene and the development of vaccines and antibiotic therapies, physicians began to realize that not all people with infections died, and with increased life span, those with recurrent infections were recognized.

In the second half of the twentieth century, the occurrence of unusual (opportunistic) infections led to the understanding of individual susceptibility to infection. Furthermore, increased susceptibility seemed to occur in some members of a given family, but not in others. Hence, the realization that in some instances, susceptibility was inherited and this led to the discovery that defects in immune mechanisms resulted in pathologies known as PIDs. However, the effective action of antibiotics can also “mask” the recognition of PIDs. When an infection occurs and resolves, not all doctors will suspect a PID. So many patients have a long history of infections before a PID diagnosis is made and chronic sequelae have already developed (bronchiectasis, malabsorption, etc.). Since PIDs are chronic diseases, once recognised, patients require specific care for the rest of their lives.

### Need for awareness: Role of key stakeholders

Greater awareness is key to ensure PID patients can be diagnosed, treated, and lead productive lives all over the world. Target groups include the public, patient, and healthcare professionals, especially primary care physicians and specialists to whom PID patients may present – see Section “Support for PID Diagnosis and Complications in Other Medical Specialties.” Whilst awareness of PIDs has been increasing dramatically in certain regions of the world, the fact is that today a large majority of PID patients remain undiagnosed and do not have access to appropriate treatment.

Increasing awareness is of utmost importance to ensure that the principles of care set out in this paper can be implemented as widely as possible. The nature of awareness campaigns will vary depending on the country. In many developing countries, greater medical awareness is needed; political and financial factors play a key role in educational efforts aimed at the medical and nursing communities, which are crucial to ensure the first steps are taken to improve the situation (10).

Awareness of the public is also important. In 2010, a worldwide awareness campaign was created with the launch of World Primary Immunodeficiencies Week (WPIW), which seeks to drive such awareness. Patient organizations have been working tirelessly on awareness campaigns to inform and educate the general public, health policymakers and decision makers, schools and families about PIDs in order to improve diagnosis rates and optimize access to treatment.

### Importance of access to early diagnosis and specialist care

Access to specialist care is a problem in many countries. Diagnosis of PIDs is often delayed, resulting in unnecessary complications, and appropriate management is suboptimal or even unavailable, especially in less developed regions. Access to early diagnosis and specialist care ensures the best health outcomes not only for the individual but also for society.

Early diagnosis shortens diagnostic delay that is distressing to the family, damaging to the patient and wasteful of health-care resources (11). Before the diagnosis is made, an individual suffering from recurrent bouts of infections, autoimmune, or inflammatory disease due to PIDs is often investigated by many different specialists but without appropriate treatment or management. The end result is deterioration of the patient’s condition, inappropriate use of health resources, and a feeling of helplessness among all parties. The data resulting from a new service to over 1,000 suspected PID patients in Asia showed that families often lost one or more children from an undiagnosed PID before the current child was diagnosed (Pamela Lee, personal communication). Precise diagnosis can lead not only to specialist care for the patient but also to genetic counseling/prenatal diagnosis for future children.

Unfortunately, PID specialist care is often absent in less developed regions, even once an accurate diagnosis of PID is made. Many governments, even in fast-growing economies, do not fund life-long Ig replacement or HSCT, though both therapies are effective and lifesaving. HSCT is a one-off curative treatment for patients with several PIDs. Hence, access to early diagnosis must be coupled with access to specialist care, to ensure appropriate therapy. This is a financial, and initially also a technical, challenge for many countries, which require at least temporary international collaborations to provide the necessary treatment, especially for HSCT. Therefore governments should recognize established reference centers and work in collaboration with them.

## Principle 1: The Role for Specialized Centers

### Diagnostic facilities

#### Patient diagnoses

Although it is estimated that about 70% of PID patients are undiagnosed, even in countries with existing PID facilities, it is impossible to know (12). Prompt PID diagnosis results in better use of health facilities (13) and has been demonstrated to result in lower healthcare costs overall (14).

Criteria for fast and reliable PID diagnoses are given in Table 1. Several multi-stage diagnostic protocols are available, according to the clinical presentation that will optimize speed of diagnosis and referral for therapy (2, 15).

**Table 1:** **Criteria for fast and reliable PID diagnoses**.

1. Early recognition of clinical manifestations suggestive of a PID before serious complications compromise the patient’s health
2. High-profile medical awareness of PIDs and information campaigns for referral of the patient
3. Consensus on basic screening tests, available to all primary health care and hospital doctors (i.e., complete blood count and differential; quantification of serum Ig levels)
4. Immediate access to a PID specialist for confirmation of diagnosis and speedy treatment
5. Standardization of immunological diagnostic protocols (immunophenotypes, protein analyses, *in vivo* and *in vitro* functional tests) and validation of clinical and laboratory biomarkers for predicting complications
6. Access to genetic counseling for the patient’s family after diagnosis

#### Newborn screening

Severe combined immune deficiency (SCID) is a rare group of disorders and is characterized by inadequate T lymphocyte production or severe abnormalities of function (16). SCID is fatal due to overwhelming infection(s) in the first year of life unless definitive treatment with stem cells from a healthy donor’s bone marrow or blood or gene therapy can be used to correct the underlying immune defect. In most cases, infants already have serious infections at diagnosis; if these do not respond to standard treatments, the infants die before immune reconstitution can even be established. The key to improving the outcome for SCID is early diagnosis and treatment, so that severe infections can be prevented (17, 18). This significantly improves HSCT results, with an overall survival rate above 90% in those infants diagnosed at birth due to a positive family history of SCID, compared to 40% in cases diagnosed later because of serious infection or significant complications (7). Diagnosis at birth means that babies can be both protected from infection and transplanted earlier in a better clinical state, all of which improves the chances of survival.

Newborn screening for SCID and other conditions with very low T-cell numbers can now be performed by detecting markers of healthy T-cell development in DNA extracted from the dried blood spots already routinely obtained from infants by a simple heel prick in the first few days of life (19). Results from recently established programs demonstrate that newborn screening dramatically improves the outcomes of infants with SCID (20–22) and should be implemented widely, not least as it is cost-effective.

### Specialist centers and networks for patient management

Patients with PIDs should be managed in regional specialist PID centers (see Table 2) to enable equitable geographical access to medical and nursing expertise in these diseases. Formal links should exist between these regional immunology centers, with recognized referral pathways for treatments. National centers in different countries will vary depending on geography, available resources and expertise but all should reach internationally agreed standards of care, as in other rare diseases (23).

**Table 2:** **Criteria for regional specialist PID centers for adults/children**.

• Meet professionally defined minimum standards for PID diagnostic and treatment services
• Provide specialist diagnostic and management services for patients within an appropriate catchment area and to be accessible to this population
• Provision of HSCT for children nationally and internationally
• Have effective patient engagement and monitor the patient experience regularly to inform improvement in practice
• Ensure effective integrated care with primary and secondary healthcare services, in particular, integrating with other hospital specialties (see Table 3)
• Commit to training and professional development for sustainability
• Contribute to national and thence international PID patient registries (see National Registries)
• Undertake PID research
• Contribute to organization/lead the local/national network
• Undertake primary responsibility for clinical and observational trials in PIDs

A national professional network raises standards of care through dissemination of guidelines, registration, and peer review of PID centers, patient registries, and professional leadership in PID (24). Established professional networks, for which there are different models in different countries, could be adopted as the basis for provision of PID management by national healthcare providers, whether governmental or insurance companies.

### Support for PID diagnosis and complications in other medical specialties

The immune system is distributed throughout the entire body. Accordingly, disturbances of immune function will have repercussions on all other organ systems and patients may present to a variety of medical and pediatric specialists. In addition, clinical immunologists (adult and pediatric) cannot have the necessary expertise to manage all potential medical and social complications and must therefore have access to a broad range of supporting specialist services, with which they have good working relationships and mutual understanding of the problems that are particular to PID patients. The importance of access to biopsy services and microbiological investigations cannot be over emphasized in all specialties. These services (listed in Table 3) should ideally be in the same institution, to facilitate consultation and be accredited to appropriate national/international standards.

**Table 3:** **Key disciplines related to PID services**.

• High-quality laboratory diagnostic services including for the investigation of suspected PIDs
• Access to named histopathologists familiar with lymphoid and infectious pathologies in PIDs
• Radiology services providing HR-CT, MRI, and PET scanning
• Intensive care services for management of overwhelming sepsis, unstable pediatric/adult patients after HSCT, and life-threatening complications
• Infectious diseases, to assist with the diagnosis and management of unusual complex infections
• Respiratory medicine, to diagnose and advise in the management of bronchiectasis and interstitial lung diseases
• Clinical hematology and hematological oncology, to undertake diagnostic bone marrow examination; to manage severe cytopenias, stem cell transplantation in children and adults, lymphoma and leukemia treatment
• Gastroenterology (adult and pediatric), for the management of malabsorption, bowel infection, and other recognized complications (autoimmune/inflammatory bowel disorders). This must include endoscopy facilities and dietician support
• Hepatology, to diagnose and treat recognized hepatic complications; this may include consideration of liver transplantation
• Dermatology, to diagnose autoimmune and infective skin conditions particular to PIDs
• Clinical genetics, to help diagnose complex PID syndromes and to counsel affected families
• Other tertiary services may also be needed, for example, otorhinolaryngology (ENT), ophthalmology, neurology, and neurosurgery, as well as social services and psychiatric support
• All services should participate in clinical audit of their involvement of PID services

### Adolescent care

Transition from adolescence to adulthood is characterized by changes of many kinds: physical, social, psychological, educational, and domestic (25). Coping with a chronic condition as well can make adolescence more complicated.

Pediatric patients are usually treated from an early age by the same staff, making it more difficult for the parent or patient to trust new faces. Without a defined and coordinated pathway guiding them toward adult services, the adolescent patient can become lost to the system, leading to poor compliance with treatment, potential irreversible organ damage, lower life expectancy, and reduced quality of life, all of which have health and cost implications.

As in other chronic diseases, planned transitional care for PID patients is often overlooked but is a cost-effective way of preventing poor compliance resulting in further damage. It is vital to ensure easy access to essential healthcare and support during this period.

### Role of new genetics

There are currently over 230 genes that, when mutated are known to disrupt immune function, and so cause a PID. This list is rapidly expanding with the detection of more genes that are proving to be important in explaining disorders of immune regulation and autoimmunity as well as defects of infection protection.

Recent advances in genetic technology have helped immensely in the diagnosis of PIDs. These include traditional sequencing of specific genes (particularly in families for genetic counseling), whole exome (and soon whole genome) sequencing (both with appropriate bioinformatics for interpretation) and chromosomal microarrays (detailing which parts of the genome are missing or duplicated). Another important area of investigation is the “mutational load” of genetic lesions causing PIDs, which is the combined impact of mutated genes, and copy number variations, which cause missing or duplicated parts of genes or entire genes (26). The latter has been especially relevant in common variable immunodeficiency disorders (CVID) (27), the commonest form of symptomatic PID. Future studies will likely show that regulatory regions of DNA are also important for expression of immune system genes and changes involving genes during life – epigenetics – may also be involved. Activating mutations can cause PIDs and somatic mutations may require analysis of DNA from particular cell populations to establish a diagnosis.

These technologies are already available in some centers and will be increasingly used in patient care in all medical specialties. As costs tumble and access to these technologies becomes more widespread, a combination of techniques will be used to diagnose previously unknown PIDs as well as provide earlier detection, with prognostic, therapeutic, and genetic counseling benefits.

## Principle 2: The Importance of Registries

### National registries

Primary immune deficiencies are recognized as rare conditions and data on epidemiology of PIDs is scarce, although many countries across the EU and worldwide have implemented registries for PIDs. An example is that established in France in 2005; the Reference Center for PIDs (CEREDIH) (28) runs the largest national PID registry worldwide, with dedicated and highly trained staff. It is based on a tight network of all university teaching hospitals, with 130 clinicians and at least 30 diagnostic immunology laboratories (29). National registries are important tools for assessing the proportion of affected individuals among the general population (prevalence) as well as measuring the number of new cases diagnosed each year (incidence), detection of areas of low-diagnostic rates and provision of insights on diagnostic delay associated with increased morbidity and mortality. A registry also provides information that is helpful to governments regarding estimates of those not diagnosed, to aid planning of educational programs and provision of treatments and their costs. Presentation of this data to pharmaceutical industries helps to ensure that the supply of relevant medical products meets demand.

Thus, a national registry is an important tool for health policy makers, stakeholders, and health insurers, enabling plans for allocation of therapies and the development of innovative treatments, as also demonstrated in the UK Demand Management Plan (30).

### International experience of registries

Studies on PIDs require international collaboration. Even in large individual European countries, no more than six to eight new patients with particularly rare genetic variants of PIDs are diagnosed per year. To collect information on all PIDs including the very uncommon cases, the European Society for Immune Deficiencies (ESID) set up an initial registry in 1994 in Sweden, supplemented by an online database in 2004. In the last 2 years, the ESID registry has been completely revised in order to provide information for national registries, healthcare providers and the European Commission. Evaluation of large registry data also provides information for individual physicians, with limited personal experience of PIDs, to answer pressing questions relating to patients or families (31).

Other continents have followed with the formation of continental registries by the Latin American Society for Immune Deficiencies (LASID) and more recently the African Society for Immune Deficiencies (ASID), and others are planned. These registries also have a role in raising awareness in countries in which the only immunodeficiency receiving attention from healthcare providers and the general public is HIV – a totally unrelated condition.

The United States Immune Deficiency Network (USIDNET), a program of the Immune Deficiency Foundation (IDF), was created in 2003 for patients in Canada and the US. The aim was to solicit, develop, evaluate, and implement clinical research strategies to advance detection, understanding, diagnosis, and treatment of PIDs. The USIDNET registry records patient data to provide both support for retrospective research studies and a source of help to physicians in making clinical decisions. USIDNET is also utilized by patients seeking information on PIDs and engages them holistically (32, 33).

International registries also provide information to centers in developing countries in which there are no national networks as yet.

## Principle 3: The Need for International Collaborations for Scientific Research

International collaboration is not only important for diagnosis (genetics) and treatment (HSCT) of patients but also for research. Clinical research on PIDs faces a range of difficulties, because of scarcity of these diseases. International collaboration is necessary in order to collect sufficient patient numbers for adequate clinical research, as well as to develop new diagnostic and therapeutic strategies, for epidemiologic studies and the identification of novel disease-associated genes (34). PID disorders are caused by a large variety of genetic defects, and not all monogenetic defects have been discovered, to date, let alone those that are oligogenic or disease modifying genes. Worldwide networks, such as the JMF Network (35), need to be encouraged and developed in all countries and continents in which these support facilities do not yet exist. It is important to note that the EU, among others, has created special programs for rare diseases (23), which help to fund the necessary financial coverage for this type of research. The International Rare Diseases Research Consortium (36) provide general guidelines for diagnostic investigations, encouragement for drug/treatment development, visibility for funding calls, and publication of new results.

## Principle 4: The Role of Patient Groups

Patient organizations have a significant role to play in healthcare systems and are increasingly key stakeholders in the political decision-making processes in healthcare. Today, it is well recognized that patient representatives have become true experts in their conditions and relevant treatments, and can bring unique and personal perspectives on the impact of diagnosis and treatment to their communities. All countries should aim to have an efficient national patient organization, representing all PID patients – children and adults – in order to give them a voice and represent their interest in policymaking. Effective national patient organizations provide advice, education, and support to patients and families and their healthcare providers too. They have a pivotal role to play in ensuring that their community is kept informed and updated about latest developments in a wide range of areas including medical and scientific advances, political and regulatory decisions, supply and safety of life-saving treatments.

Patient groups are increasingly active in collecting clinical data and participating in the management of registries, which yield important useful information and help to guide decisions affecting their health. They are also the driving force behind awareness campaigns around the world. To do this successfully, collaboration with doctors and other key stakeholders such as nurses, regulators, civil servants, decision makers, and industry is of vital importance, as many voices resonate louder than one. Patient-centered policies improve compliance, and therefore outcomes, once the interest of the patient is at the center of the decision making process.

At the international level, organizations such as the International Patient Organisation for Primary Immunodeficiencies (IPOPI) (37) have 51 national patient member organizations so far (38) and are actively involved in assisting new countries to start such an organization. They are involved on many different fronts, providing the patients’ expertise in international committees and institutions, including making representations to the European institutions and the World Health Organization.

## Principle 5: Management and Treatment Options for PIDs

### Nature of Ig replacement therapies

There are several types or “classes” of human Ig in blood; these include IgG, IgA, and IgM. Among these, IgG has the highest concentration in blood and body fluids and is critical for protection against infection. IgG is purified safely from human plasma and administered to people who are unable to produce a sufficient amount of good quality of IgG themselves, as a result of PID. Such IgG replacement treatment is literally life saving for such individuals and usually needs to be continued for life.

The IgG products used for replacement therapy are protective against a broad range of infections. This explains why therapeutic Ig cannot be made by recombinant technology, as for other drugs. Ig therapy is also distinct from “monoclonal antibodies” that have recently been developed to treat specific diseases rather than to protect from all types of infections.

Immunoglobulin replacement therapy is absolutely essential for the treatment of the majority of patients with a PID. There are a variety of IgG products currently marketed worldwide (39). Each new product undergoes clinical trials to establish efficacy (protection from infection), safety (no disease transmission or seriously harmful adverse events), and tolerability (minimal side effects) before it can be sold.

Although Ig therapies have been in clinical practice for over 60 years, there are still challenges since Ig products are not yet available to all PID patients (Table 4).

**Table 4:** **Current challenges to Ig therapy**.

• Provision of finances to ensure availability of several Ig products in every country, to enable wide access to appropriate therapies, as per WHO Essential Medicines Lists
• Early diagnosis to prevent infection-related complications such as bronchiectasis
• Selection of optimal therapy and dosage for each patient, with regular medical follow-up to check reduction/abolition of breakthrough infections
• Increasing doses with growth in children
• Expert treatment centers, with dedicated nursing staff, to avoid side effects due to incorrect infusion techniques in first few infusions
• Training for self-infusion by suitable patients at home, with regular follow-up to ensure on-going high standards
• PID patients are prioritized for Ig products in times of restriction (for financial or availability reasons)
• Improvement of outcomes for complex patients by using additional therapies for disease-related complications

#### Safety of immunoglobulin therapies

Immunoglobulin products are prepared from plasma collected from tens of thousands of screened blood/plasma donors under carefully controlled conditions and thus contain a vast repertoire of neutralizing antibodies to defend the recipient against the range of pathogens. Each company uses slightly different proprietary methods to purify IgG molecules to a high degree (typically >98%) and to reduce the possibility of disease transmission. All available products have been shown to protect against infections.

Over the last 50 years, purification and manufacturing methods have improved tremendously. There have been no known cases of disease transmission by therapeutic Ig in the past 20 years. Since 1994, viral safety has been robust, due to specific genetic testing of individual donations and implementation of a number of viral inactivation/removal steps. Authorized products contain at least two viral inactivation/removal steps. To date, there has been no known transmission of pathological prions (the agent causing variant Creutzfeldt–Jakob-disease) with Ig.

Most PID patients are able to receive Ig replacement therapy without side effects at all. A few patients may need some medicines (non-steroidal anti-inflammatory drugs and antihistamines) to treat mild side effects. A very few (1%) may have more troublesome side effects, but serious reactions are very rare indeed. With the variety of products and infusion options available, almost all patients can be treated successfully with Ig. However, it is important to realize that there is no single Ig product or method of administration that is suitable for all PID patients.

All blood products carry an infection risk, and like others, Ig products are regulated by national or international regulatory bodies to be sure that they are safe and of high quality. These bodies share their information worldwide. Excipients, preservatives, pH, IgA content, and protein concentration vary between Ig products and thus alter the side-effect profiles; respective warnings are stated in the product specific labeling. Constant vigilance is necessary to detect any new clusters of side effects of Ig therapy that may result from changes in manufacturing, concentration of the product, infusion rates, or expansion to new indications. Recent examples of investigations following unexpected side effects include the risk of thrombotic events and hemolytic episodes.

#### Optimal treatment levels of Ig replacement therapy

The key principle goal of Ig replacement therapy is to prevent bacterial infections and avoid organ damage that leads to chronic disease and poor quality of life. Infection prevention rather than a targeted serum IgG level is the goal of Ig replacement therapy as the protective serum IgG level varies with individual patients (40). In a meta-analysis of 17 clinical trials, there was a 27% decrease in the incidence of pneumonia for every 100 mg/kg/28 days increase in the dose of Ig therapy (41). Patients with chronic gut disease or bronchiectasis require higher doses of Ig replacement therapy (29). Other studies of patients with antibody deficiency receiving subcutaneous Ig replacement therapy show similar findings. However, cost implications may prevent optimal doses being used and it is important for patient groups and healthcare professionals to unite to explain to healthcare providers the importance, both medically and financially, of optimal dosing.

Replacement Ig therapy has been shown to be cost-effective in preventing hospitalizations and emergency departments visits, unscheduled physician visits, expensive antibiotic treatments and missed days of school or work. These studies emphasize that the appropriate dose of Ig replacement therapy enhances quality of life measures too (42).

#### Access to a wide choice of Ig therapies

Human Ig preparations reflect the infectious or immunization exposures of the plasma donors. However, due to patent law, they cannot be considered as generic drugs (i.e., connected together by the same chemical composition, pharmacological effects, treatment use, and same adverse effects) as Ig production methods vary. Therapeutic Ig is administered by three routes: subcutaneously (SC), intravenously (IV), or intramuscularly, though the latter is no longer recommended due to high rates of infusion related reactions. SC and IV have specific advantages and disadvantages, which may depend on the patient’s medical background or on personal preferences. Frequency of treatment, the availability of good venous access and other factors all play a role and it is impossible to say that one brand or route of administration is generally better than another. Therefore, all countries and immunodeficiency centers should have access to a wide spectrum of Ig products, to provide optimal treatment for all immunodeficient patients.

#### Recognition of PIDs as priority indications for Ig therapy

Immunoglobulin replacement therapy for PIDs was primarily developed for patients with antibody deficiencies in the last half of the twentieth century. Since then many other indications in autoimmunity or immune dysregulation have been recognized, resulting in much larger amounts of intravenous Ig being used in these conditions, often in off label usage not recognized by the regulatory authorities. For some autoimmune diseases, alternative therapies are available and monoclonal antibodies, such as rituximab, may supersede the use of high dose Ig therapies in autoimmunity in time. However, the available amount of Ig is limited, depending as it does on blood donors. Therefore, for now, Ig therapy should be prioritized for PIDs since there are clear indications, proven efficacy, and no alternative treatments available. This was the reason why Ig products were accepted to the WHO Lists of Essential Medicines both for adults (43) and children (44).

### Site of Ig treatment (specialist center, local hospital, home)

To replace missing antibodies, the antibody deficiency patients need Ig on a regular basis for the duration of their lives. Although initial treatments are started under supervision in a day care facility with experienced staff, once stable, patients can either self-treat or be treated at the local hospital or health-care center. There still needs to be regular contact with the specialist immunology nurses and both easy access to and regular follow-up by the PID doctor, since complications can occur later and significant damage from infection or chronic inflammation, particularly for children or adults who are diagnosed late, needs to be monitored. Research has shown that self-infusions at home after education are effective, safe (45), and appreciated by patients and families, as this results in less disruption to work and school as well as social lives (Table 5) (31, 46). This is also appropriate for some children or infants awaiting HSCT if a donor is difficult to find.

**Table 5:** **Advantages of programs for self-infusion at home**.

• Adult patients report that they are less tired, can plan their lives and do not have to miss work to attend treatment sessions
• Parents report that home-therapy keeps the child healthier due to regular treatment, enabling participation in school activities
• Participation in family/social and leisure activities for adults and playing with friends for children allow them to feel and act like others
• Parents themselves report less worry for the future of their child, fewer restrictions or sudden changes in plans in relation to family activities (e.g., holiday trips), less tension at home and more time for own needs and therefore have a higher quality of life

### Hematopoietic stem cell transplantation

Hematopoietic stem cell transplantation (from blood or bone marrow) is the only cure for severe, otherwise fatal PIDs that present in infancy or early childhood and all SCID children should have access to this life-saving therapy, regardless of where they live.

Bone marrow resides in the cavities of the long bones of the body and contains stem cells for all the cellular components of the blood. Stem cells are capable of repopulating a new immune system throughout life, so some are present in blood as well and can be purified following adult or cord blood donation.

The first bone marrow transplants were performed over 30 years ago but until recently were too risky to do in any but the sickest patients. Today, progress has made HSCT feasible for an increasing number of patients, though outcomes are determined by a variety of factors (Table 6).

**Table 6:** **Current challenges to hematopoietic stem cell transplantation**.

• Identifying candidates before they sustain significant damage from infection, particularly for children or adults diagnosed late
• Recruiting appropriate donors, since a “match” between donor and patient is essential for a good outcome
• Improving the outcomes for the sickest patients with complex PIDs depends on determining the involvement of other tissues or organs

Tissue types are diverse around the world and statistically the chances of finding a match are higher within one’s own ethnicity. Donor recruitment strategies vary from country to country but patients who come from under-represented ethnic backgrounds can have a difficult time finding a “match.” Knowledge regarding transplantation, cultural, and technical limitations can hamper the broad recruitment needed to ensure that every patient who requires it has access to this life-saving treatment.

Raising awareness of the success of HSCT and additional research into outcomes is needed to continue the impressive successes in this field. Optimal treatment to prepare the body for the transplant has been studied for a decade, with tremendously improved outcomes (6), yet children and adults still die due to the risks of the process and patients require great stamina for the rigors of maintaining ideal conditions. Gentler strategies are under development and additional research may pave the way for our goal of curing 100% of patients who need HSCT.

### Additional antimicrobial measures

Infection is the most common presentation, the nature of which depends on the underlying immune defect. More than one infection may be present and more than one organ affected. Occult infections require careful imaging, tissue sampling, and molecular techniques needed to identify the pathogen. Treatment of infection in PID patients is complex, often requiring one or more broad-spectrum antimicrobials and for prolonged time-courses, and physiotherapy is essential for those with lung complications.

If an innate PID is diagnosed for which there is no specific effective therapy, prophylactic antimicrobials may be given. Physical barriers range from sterile positive pressure ventilation for extremely immunodeficient infants to specific measures such as boiling water or avoidance of exposure to fungi. Anti-bacterial prophylaxis is used to prevent pneumococcal or meningococcal disease in patients with complement deficiency or hyposplenism. Treating existing fungal infections in patients with innate deficiencies is a huge challenge, with larger doses of increasingly toxic antifungal agents and even neutrophil transfusions as well as surgical drainage.

### Immunological and other treatments

Other replacement therapies or even immunosuppression to counter the aberrant immune responses may be necessary. Examples include granulocyte-colony stimulating factor (G-CSF) to boost the production of neutrophils or gamma interferon in patients with defective neutrophils.

Long acting adenosine deaminase (PEG-ADA), an enzyme replacement therapy, is administered to patients who lack ADA prior to HSCT.

Anti-inflammatory agents, such as corticosteroids, and immunosuppressive agents are useful for particular complications (including respiratory, gastro-intestinal, and dermatological). Nutritional supplements and other types of therapies (physiotherapy, psychotherapy) are also used to treat PID patients with specific complications.

This list of other treatments is not exhaustive but provides examples of the complexity of therapies that should be available to ensure appropriate care for all PID patients.

### Gene therapy

A new approach to replacement of faulty genes has been developed in recent years, though currently remains a research tool. Gene therapy is defined as the addition of a normal copy of a gene to a patient’s purified stem cells to supplement the one that is defective or absent. This is currently reserved for children for whom HSCT is not available (generally because they do not have a suitable matched donor for HSCT). Successful clinical trials started in Paris in 1999 for patients with X-linked SCID. More recently, these have been extended at several centers to include patients with ADA SCID, CGD, and Wiskott–Aldrich syndrome. Most of the patients have been successfully treated and are at home, off all therapy, thus, proving that such therapy is possible and effective (8).

However, there have been a small number of patients who developed leukemia-like disorders as a result of unwelcome mutations in the cells as a result of using the original retroviral vectors – a process known as insertional mutagenesis. New improved self-inactivating retroviral and lentiviral vectors have been developed incorporating additional safety features and these are now being used in clinical trials in the same patient groups.

### Vaccines

Vaccination – or more commonly – immunization is the administration of material derived from infectious material in order to produce a protective immune response to a specific pathogen (bacteria or virus) without developing the infection. The material in the vaccine can be modified (attenuated) or killed (inactivated) whole micro-organisms, defined components of the microorganism or specific proteins such as modified toxins (known as toxoids). Those that consist of live-attenuated bacteria or virus can undergo reversion to a more virulent form and cause disease (unlike the killed micro-organisms). In general, attenuated vaccines including BCG and rotavirus, must be avoided in all the severe forms of PID (SCID, CGD, T-cell defects, etc.), as in such patients there will be no protective immune response and therefore a clear risk of developing the disease itself (47). If BCG is given in the first month of life, infants with severe PIDs or MSMD develop generalized BCG-osis that compromises HSCT and is often fatal. To postpone the age of BCG vaccine would prevent this (48). If BCG administration cannot be delayed, newborn screening is important to identify at-risk babies.

Inactivated vaccines are safe for use in most PID patients but are ineffective if there is no or only a limited immune response, depending on the type of immune failure. SCID patients are only immunized after HSCT has restored a healthy immune system. If the patient cannot produce antibodies, vaccines are ineffective though patients may produce a T-cell response that will prevent or lessen any subsequent, particularly viral, infection.

In those PIDs due to neutrophil defects, complement deficiencies and some mild forms of antibody failure, vaccination is beneficial (41) and forms part of the treatment protocol.

It is necessary to discuss the risks and benefits to the specific individual with the patient’s pediatrician or specialist immunologist as well as the patient (49). Immunization of household relatives to prevent close-contact infection, such in an influenza outbreak or to provide on-going protection in a meningitis or chickenpox outbreak, is helpful.

Killed vaccines are also used in diagnostic procedures if antibody failure is suspected; failure of specific antibody production indicates an immune defect.

### Comprehensive and holistic approach to the patient

Primary immune deficiency patients encounter a diversity of clinical manifestations during their lifetime (infections, autoimmunity, granuloma, allergy, lymphoproliferative disorders, and malignancies). Thus, expert management according to the best international standard of care is warranted and acknowledgment of the personal context of the patients. Treated patients now have an increased life expectancy but there are new and unexpected complications seen with longer life, as well as difficulties in transition to adolescence and adulthood. Thought should be given to long-term care plans and access to genetic counseling when patients consider having children of their own. As their life progresses, modifications to treatment will be needed to fit in with changing circumstances such as work, studying, travel, or pregnancy. Patients need to have assistance and choice in treatment modalities and to be able to change the route, dose and location of Ig replacement (IVIgs versus SCIgs and home versus clinic) especially as children grow.

Impacts on a patient’s life include stress, anxiety, chronic fatigue, pain, disability, fertility, and psychosocial aspects. Advice about sports to relieve complications such as bronchiectasis, safety measures for traveling (including places where safe drinking water is not easily available), avoidance of inappropriate hobbies, how to obtain health insurance, etc., should be given by trained healthcare professionals. Further studies are needed.

### Emergency medicine

Primary immune deficiency patients may need emergency treatment, so every patient should have an individually tailored plan (medical diagnosis, specific therapy, expert center contact), outlining management of emergencies common to their immunodeficiency, and ensuring access to specialist medication. If direct access to the specialist center is impractical (for example, because of distance), shared care should be sought with local direct-access staff who are informed in the care required. Emergency staff are unlikely to be familiar with management of immunodeficiency and the patient may be at-risk of delayed or inappropriate care; a 24-h contact number for specialist advice must be included in the patient’s plan. This is particularly important for patients with complement deficiencies in whom the correct emergency therapy undoubtedly saves lives.

## Principle 6: Managing PID Diagnosis and Care in all Countries

### Access to PID care World-Wide

The broad scope of PID clinical presentations presents special challenges (Table 7), especially in developing countries lacking even basic health care; infectious diseases are common and so PIDs are missed. Yet, diseases that are rarer than PIDs still achieve good diagnostic and treatment levels in low-income countries through organized efforts.

**Table 7:** **Integrated approaches for PIDs**.

• Awareness for recognition and management of PIDs through clinician education, training, and advocacy groups should be taken on, often in association with those already experienced in PIDs
• Investigation and management of PIDs should be integrated into resources allocated to the epidemics of malaria, HIV, tuberculosis, and other locally prevalent diseases
• National organizations should be established to oversee the provision of national specialist PID care centers, providing the entire spectrum of clinical and laboratory services and supporting a network of smaller centers
• International collaboration between established centers and less experienced centers to guarantee access to optimal diagnostics and possibly treatments, particularly in the case of HSCT
• A national registry for PID diagnoses must be established to provide data to healthcare providers

In the context of high prevalence of infectious diseases in developing countries (in particular, infections resulting from HIV) PIDs, which are due to intrinsic failure of the immune system, are now more likely to be exposed.

### Access to PID diagnosis

Diagnosis of PID is a challenge under any circumstances, but even more so in a resource-constrained setting of developing countries. Clinicians must be sure to select scarce (often expensive) laboratory resources judiciously to avoid an unnecessary work-up in a suspected PID. Understanding the clinical clues (Table 8) and using a small set of basic tests enable diagnosis of most PIDs enables appropriate treatment to be sought. As there are few centers and few immunologists in developing countries, it is important to set up a network, using the Internet, to discuss clinical cases and support of physicians who live far from specialized centers. International links are helpful too.

**Table 8:** **Clues toward a diagnosis of a PID**.

History	Physical examination	Investigations
Failure to thrive	Paucity of lymph nodes/tonsils	Full blood count for neutropenia, lymphopenia, or neutrophilia
Onset of serious infections, especially in infancy/early childhood	Failure of obvious inflammation or unexplained inflammation	Serum immunoglobulin levels – IgG, IgA, IgM+/−IgE
Recurrent or unusually severe infections in adults	May be enlarged spleen or liver	Flow cytometry (to define blood immune cells)
Infections with an unusual or seemingly innocuous (opportunistic) organisms	Huge lymphadenopathy	Oxidative burst tests (for neutrophil function)
Chronic, non-infectious inflammation of unknown origin, leading to severe or unusual organ damage	May be persistent rash or skin infections	Microbiological examination of tissue, fluids, stool, sputum, or alveolar washings; measure IgE
Family history of severe, persistent, unusual or recurrent infections (SPUR)	Congenital defects in other organs/systems	Imaging of affected organs

With this simplified approach, it is possible to diagnose the vast majority of common PIDs (50).

### Access to PID treatments

Best level of available PID care must be pursued, even if the full range of treatment options is not available. Knowledge must be promoted on treatment and prevention of infectious diseases with hygiene measures, nutritional support, vaccines, and antibiotics. Very often developing countries are specifically faced with important challenges with regards to relatively expensive therapies used in the treatment of PIDs, mostly because of cost constraints or a lack of expertise.

Immunoglobulin therapies are listed as Essential Medicines by the WHO (43, 44) and should be made available for PID patients in all developing countries. While HSCT has been the standard of care in developed countries for most forms of severe PIDs, it is frequently not available in developing countries though the situation is slowly improving. The cost of building appropriate facilities and of providing expert training locally may seem significant but actually allows for more efficient management of healthcare resources, as many SCID patients in developing regions currently have to travel to developed countries where the cost is higher. International collaboration and the creation of regional societies such as ESID in Europe, LASID in Latin America, and ASID in Africa have been and will continue to be pivotal in increasing PID expertise in developing countries.

## Implementation

Vast gaps in knowledge of PIDs and translation of diagnosis and management into clinical practice exist between regions, countries, and even areas. This causes unnecessary suffering and deaths, particularly in developing countries. PID facilities should be at least as good as those for other, even rarer, diseases. Services for diagnosis and treatment can be developed initially alongside facilities for diagnosis and monitoring of HIV patients or located with immunization centers. In order for the best level of available PID care to be pursued, even if the full range of treatment options may not be available, knowledge of PIDs must be promoted, and infra-structure for diagnosis and care implemented.

This involves, by stages:

- Raising awareness and understanding of the clinical clues among local healthcare workers, general physicians, and pediatricians so that patients are diagnosed quickly;
- Provision of inexpensive tests (often already available in district hospitals/healthcare centers) plus guidance on their interpretation and indications for further complex testing;
- Defining practical pathways to obtain appropriate therapies for the patient.

With this simplified approach, it is possible to diagnose and treat the majority of common PIDs.

To achieve these aims, professional stakeholders in PIDs have to come together in a network to:

- Share information informally regarding individual patients and access to all relevant treatments including HSCT, and to make this knowledge available to others;
- Agree to share relevant specialist investigations so that all useful assays are available to patients throughout every country;
- Construct and publish a consensus document on the need for and provision of PID services nationally (51) and use this to persuade healthcare providers to establish regional and national centers for complex investigations and therapies;
- Seek advice from established centers in other countries or continents and to establish links for treatment, training, and education with these centers;
- Meet at least annually and to correspond regularly or post information and questions on a website;
- Include patient organizations, who are key stakeholders in healthcare decisions and who can also assist with clinical data collection.

Established professional networks should be recognized, formalized, and adopted as the basis for provision of PID management by national healthcare providers, whether governmental or insurance companies. Network responsibilities also include registration of patients (33), setting of national standards, and auditing PID services to these standards (52).

As in the provision of other rare diseases, services for PID patients usually involve recognized expert centers for complex diagnostic facilities as well as experience in treatments, whether Ig replacement or HSCT. In well-established countries, there may be several expert centers, each concentrating on a particular type of PID, with a broad range of supporting specialist services; this has the advantage of concentrating resources for research. Good links with pediatric services are essential for planned transitional care and the importance of a national registry has been emphasized above (53).

## Conclusion

In conclusion, we call upon international and national healthcare policy makers to join us in taking strong and decisive action to ensure that people with PIDs are diagnosed as early as possible and have appropriate access to safe, efficient life-saving treatments, and optimum care throughout the world.

We endorse the above principles, as elements of PID care provision that should be available and implemented in each country. These include specialized centers, PID registries, provision for transnational research, patient organizations, access to services for diagnosis and treatment, the need for sustained access to all treatments including Ig therapies and HSCT, and important considerations for developing countries.

## Authorship

The Principles of Primary Immunodeficiency Care were led by the International Patient Organisation for Primary Immunodeficiencies (IPOPI) and developed by an interdisciplinary working group of 30 leading experts in PID care from 12 countries in 6 continents. Over a 2-year period, this group reviewed current best practice care and guidelines, as well as assessed the needs of those both providing and receiving PID care and treatment worldwide. Representatives from patient organizations and specialist immunology nurses were included.

## Conflict of Interest Statement

The authors declare that the research was conducted in the absence of any commercial or financial relationships that could be construed as a potential conflict of interest. The Review Editor Klaus Warnatz declares that, despite having collaborated on a publication in the last 2 years with the authors Helen Chapel and Hubert Bobby Gaspar, the review process was handled objectively.
